# Comparison of abdominal adiposity and overall obesity in relation to risk of small intestinal cancer in a European Prospective Cohort

**DOI:** 10.1007/s10552-016-0772-z

**Published:** 2016-06-13

**Authors:** Yunxia Lu, Amanda J. Cross, Neil Murphy, Heinz Freisling, Ruth C. Travis, Pietro Ferrari, Verena A. Katzke, Rudolf Kaaks, Åsa Olsson, Ingegerd Johansson, Frida Renström, Salvatore Panico, Valeria Pala, Domenico Palli, Rosario Tumino, Petra H. Peeters, Peter D. Siersema, H. B. Bueno-de-Mesquita, Antonia Trichopoulou, Eleni Klinaki, Christos Tsironis, Antonio Agudo, Carmen Navarro, María-José Sánchez, Aurelio Barricarte, Marie-Christine Boutron-Ruault, Guy Fagherazzi, Antoine Racine, Elisabete Weiderpass, Marc J. Gunter, Elio Riboli

**Affiliations:** Department of Epidemiology and Biostatistics, School of Public Health, Imperial College London, Norfolk Place, London, W2 1PG UK; International Agency for Research on Cancer (IARC-WHO), Lyon, France; Cancer Epidemiology Unit, Nuffield Department of Population Health, University of Oxford, Oxford, UK; Department of Cancer Epidemiology, German Cancer Research Center (DKFZ), Heidelberg, Germany; Department of Surgery, Skåne University Hospital, Lund University, 205 02 Malmö, Sweden; Department of Odontology, Umeå University, Umeå, Sweden; Department of Clinical Sciences, Genetic and Molecular Epidemiology Unit, Skåne University Hospital Malmö, Malmö, Sweden; Department of Biobank Research, Umeå University, Umeå, Sweden; Dipartimento Di Medicina Clinica E Chirurgia, Federico II University, Naples, Italy; Epidemiology and Prevention Unit, Fondazione IRCCS Istituto Nazionale dei Tumori, Via Venezian, 1, 20133 Milan, Italy; Molecular and Nutritional Epidemiology Unit, Cancer Research and Prevention Institute ISPO, Florence, Italy; Cancer Registry and Histopathology Unit, “Civic - M.P. Arezzo” Hospital, ASP Ragusa, Italy; Department of Epidemiology, Julius Center for Health Sciences and Primary Care, University Medical Center Utrecht, Utrecht, The Netherlands; Department of Gastroenterology and Hepatology, University Medical Centre, Utrecht, The Netherlands; Department for Determinants of Chronic Diseases (DCD), National Institute for Public Health and the Environment (RIVM), Bilthoven, The Netherlands; Department of Social and Preventive Medicine, Faculty of Medicine, University of Malaya, Kuala Lumpur, Malaysia; Hellenic Health Foundation, 23 Alexandroupoleos, 115 27 Athens, Greece; Bureau of Epidemiologic Research, Academy of Athens, 13 Kaisareias Street, 115 27 Athens, Greece; Department of Hygiene, Epidemiology and Medical Statistics, University of Athens Medical School, 75 M. Asias Street, Goudi, 115 27 Athens, Greece; Unit of Nutrition, Environment and Cancer, Cancer Epidemiology Research Program, Catalan Institute of Oncology, Av. Gran Via 199-203, 08908 L’Hospitalet de Llobregat, Spain; Department of Epidemiology, Murcia Regional Health Council, IMIB-Arrixaca, Murcia, Spain; Consortium for Biomedical Research in Epidemiology and Public Health (CIBER Epidemiología y Salud Pública-CIBERESP), Madrid, Spain; Department of Health and Social Sciences, Universidad de Murcia, Murcia, Spain; Escuela Andaluza de Salud Pública, Instituto de Investigación Biosanitaria ibs.GRANADA, Hospitales Universitarios de Granada/Universidad de Granada, Granada, Spain; Navarre Public Health Institute, Pamplona, Spain; Inserm, Centre for Research in Epidemiology and Population Health (CESP), U1018, Nutrition, Hormones and Women’s Health team, 94805 Villejuif, France; Université Paris Sud, UMRS 1018, 94805 Villejuif, France; Institut Gustave Roussy, 94805 Villejuif, France; Department of Community Medicine, Faculty of Health Sciences, The Arctic University of Norway, University of Tromsø, Tromsø, Norway; Department of Research, Cancer Registry of Norway, Oslo, Norway; Department of Medical Epidemiology and Biostatistics, Karolinska Institutet, Stockholm, Sweden; Department of Genetic Epidemiology, Folkhälsan Research Center, Helsinki, Finland

**Keywords:** Abdominal obesity, Obesity, Cancer, Small intestine

## Abstract

**Background:**

The etiology of small intestinal cancer (SIC) is largely unknown, and there are very few epidemiological studies published to date. No studies have investigated abdominal adiposity in relation to SIC.

**Methods:**

We investigated overall obesity and abdominal adiposity in relation to SIC in the European Prospective Investigation into Cancer and Nutrition (EPIC), a large prospective cohort of approximately half a million men and women from ten European countries. Overall obesity and abdominal obesity were assessed by body mass index (BMI), waist circumference (WC), hip circumference (HC), waist-to-hip ratio (WHR), and waist-to-height ratio (WHtR). Multivariate Cox proportional hazards regression modeling was performed to estimate hazard ratios (HRs) and 95 % confidence intervals (CIs). Stratified analyses were conducted by sex, BMI, and smoking status.

**Results:**

During an average of 13.9 years of follow-up, 131 incident cases of SIC (including 41 adenocarcinomas, 44 malignant carcinoid tumors, 15 sarcomas and 10 lymphomas, and 21 unknown histology) were identified. WC was positively associated with SIC in a crude model that also included BMI (HR per 5-cm increase = 1.20, 95 % CI 1.04, 1.39), but this association attenuated in the multivariable model (HR 1.18, 95 % CI 0.98, 1.42). However, the association between WC and SIC was strengthened when the analysis was restricted to adenocarcinoma of the small intestine (multivariable HR adjusted for BMI = 1.56, 95 % CI 1.11, 2.17). There were no other significant associations.

**Conclusion:**

WC, rather than BMI, may be positively associated with adenocarcinomas but not carcinoid tumors of the small intestine.

**Impact:**

Abdominal obesity is a potential risk factor for adenocarcinoma in the small intestine.

## Introduction

Small intestinal cancer (SIC) is very rare, with an annual incidence rate ranging from 0.2 to 2.6 per 100,000 people per year worldwide [1–4]. Although the small bowel comprises more than two-thirds of the length of the digestive tract and more than 90 % of its mucosal surface area [5], <5 % of all gastrointestinal tract cancers and <1 % of all cancers arise in the small intestine. In autopsy series, however, the rate of SIC is much higher (4–5 % of malignant neoplasms), indicating either a low detection rate during the patients’ lifetime or a relatively high proportion of non-aggressive cancers [6]. Recent studies from the USA and Europe have indicated an increasing incidence of SIC, which seems to be explained by the increasing incidence of adenocarcinoma of the duodenum [1, 3, 4, 7–11]. The main histological subtypes of malignant SICs include adenocarcinomas, carcinoid tumors, lymphomas, and sarcomas. Adenocarcinoma is the dominant histological subtype in the duodenum, and carcinoid tumors are most common in the ileum [12–15].

The etiology of SIC is largely unknown, with few epidemiological studies conducted so far. Age, sex, race/ethnicity, dietary factors, smoking, alcohol consumption, and reproductive factors have been examined in relation to SIC in several case–control studies [16–20] and population-based or registry-based cohort studies [21–24], but the results have generally been inconsistent. Two registry-based studies from Sweden and the USA have suggested that obesity may be positively associated with SIC [24, 25]. These studies used clinical diagnosis of obesity rather than body mass index (BMI), which may not reveal the true extent of the association between obesity and SIC because patients with a clinical diagnosis are generally very obese (BMI > 35 or 40 kg/m^2^, depending on country) [26]. A high BMI has been indicated to be positively associated with SIC in three cohort studies [21, 27, 28], but the association was limited to carcinoid tumors [21] or men [27], or the association was not statistically significant [28]. Conversely, a case–control study from Italy found that a lower BMI was associated with increased risk of adenocarcinoma of the small intestine [20], although assessing the association between BMI and cancer in a case–control setting is problematic because of the potential for reverse causality. No previous studies have reported on abdominal obesity and SIC risk, which is an established risk factor for other gastrointestinal malignancies, such as colorectal cancer [29]. Using data from the European Prospective Investigation into Cancer and Nutrition (EPIC), one of the largest studies to date with systematically and extensively measured anthropometric data, the current study investigates overall obesity and abdominal obesity in relation to incident SIC.

## Materials and methods

### Study population

Detailed information on the design and data collection in the EPIC study was described previously [30, 31]. In brief, EPIC is an ongoing multicenter prospective cohort designed to investigate the associations between diet, anthropometry, lifestyle, genetic and environmental factors, and various types of cancer and other chronic diseases. In total, 521,330 men and women from 23 study centers in ten European countries (Denmark, France, Germany, Greece, Italy, Norway, Spain, Sweden, The Netherland, and UK) were recruited between 1992 and 2000. After exclusion of participants who were lacking questionnaire information (*n* = 45,198), missing values for waist circumference (WC) or hip circumference (HC) (*n* = 115,381), and missing data for smoking, education, and physical activity (*n* = 21,985), our analytic cohort consisted of 338,766 men and women.

At baseline, detailed questionnaires were administered, anthropometric measurements were carried out, and biological samples were collected. The cohort participants have been followed over time through the inspection of medical records or/and through tumor registry linkage and/or active follow-up. Written informed consent was provided by all participants, and ethical approval for the EPIC study was provided from the review boards of the International Agency for Research on Cancer (IARC) and local participating centers.

### Identification of SIC cases

All participants were followed over time for the occurrence of cancer and other diseases, as well as for overall and cause-specific mortality. Incident cancer cases were identified by follow-up based on population cancer registries (Denmark, Italy, Netherlands, Norway, Spain, Sweden, and UK) and other methods such as health insurance records, pathology registries, and active contact of study subjects or next of kin (France, Germany, and Greece). For self-reported information provided by the participants or their next of kin, the potential cases were thereafter verified by physician records. The tenth version of the International Classification of Diseases (ICD-10) and the second revision of the International Classification of Disease for Oncology (ICDO-2) were used to code SIC by anatomical location (ICD10: C17) [32, 33]. Histological subtypes included adenocarcinoma (morphology codes: 8140/3, 8141/3, 8143/3, 8480/3, 8481/3, 8144/3, 8210/3, and 8211/3), malignant carcinoid tumors (morphology codes: 8240/3, 8241/3, 8244/3, 8245/3, and 8246/3), lymphomas, and sarcomas, although there were too few of the latter two to separate as single groups. Subjects were considered to be at risk from their enrollment into the cohort until diagnosis of SIC, death, censoring (e.g., loss to follow-up, emigration, diagnosis of other malignancies), or end of follow-up, whichever occurred first.

### Assessment of anthropometric data

Body weight (kilograms, kg) and height (centimeters, cm) were measured without shoes according to standardized procedures. WC (in cm) was measured either at the narrowest circumference of the torso or at the midpoint between the lower ribs and the iliac crest according to study center, except in Norway and Umeå (Sweden), where WC was not assessed. HC (in cm) was measured horizontally at the level of the largest lateral extension of the hips or over the buttocks. To account for between-center heterogeneity in anthropometric measurement methods, participants who had measurements taken while normally dressed had 1.5 kg subtracted from weight and 2.0 cm subtracted for WC, and participants who were measured in light clothing had 1 kg subtracted from weight.

BMI was calculated as body weight (in kg) divided by height (m^2^). Waist-to-hip ratio (WHR) and waist-to-height ratio (WHtR) were calculated from measurements of WC, HC, and height. In the “Health-conscious” group in the UK (these participants were recruited by post), self-reported anthropometric data were adjusted using prediction equations derived from a subset of participants with both self-reported and measured anthropometric data available [34].

### Statistical analysis

Hazard ratios (HRs) and 95 % confidence intervals (95 % CIs) for the associations between anthropometric measures and SIC were estimated using Cox proportional hazard models, stratified by sex and country. Age was used as the underlying timescale in the Cox model. Anthropometric indices were analyzed based on continuous and categorical variables, but due to the small number of cases, we only reported the results based on continuous variables.

For each anthropometric indicator, we analyzed the data based on a crude model adjusted for age and stratified by sex and country and a multivariable model. For the multivariable model, we selected potential confounders based on two approaches. First, we chose confounders based on previous etiological studies on SIC or colorectal cancer. Secondly, we used stepwise selection where significance level (alpha) for entry and retention in the model were both set at 0.2. Combining the first and the second approaches, we included the following covariates in the multivariable model: age (in 1-year categories), sex (male and female), country (categorical variable for countries included in EPIC), education (none/primary school, technical/professional, secondary, and longer education), smoking status and intensity (never; current, 1–15 cigarettes/day; current, 16–25 cigarettes/day; current, 26+ cigarettes/day; former, quit smoking ≤10 years; former, quit smoking 11–20 years; former, quit smoking 20+ years; and missing), baseline alcohol drinking (continuous), and physical activity (inactive, moderately inactive, moderately active, and active), defined by the Cambridge index [35]. In addition, we examined dietary variables by including a diet score (the modified Mediterranean diet score) in the multivariable model; however, this did not materially affect the findings and therefore was not included in the final models. Since the results from the crude models and from the multivariable models did not change materially, we only reported the results based on multivariable models for stratified analyses. The variable for WC was scaled to examine the effect per 5-cm increments (original value of WC divided by 5). WHR and WHtR were multiplied by 100 in the model to decrease the significant fluctuation in the small values and were interpreted as percent changes. Analyses of BMI were conducted with and without inclusion of WC, or with adjustment for the residuals of WC. The latter approach aims to reduce the influence of potentially high collinearity among these anthropometric indices [36]. The data for WC, HC, WHR, and WHtR were examined both with inclusion and without inclusion of BMI (continuous) as described by Pischon et al. [26] or by calculating residuals of the aforementioned variables when adjusted for BMI. In the model of BMI and WC residuals, the biological meaning of BMI would represent overall body fatness, while WC residuals would represent central obesity adjusted for overall adiposity. Since the results adjusted by residuals did not change materially, we did not report them in the manuscript. Further analyses were stratified by sex, BMI (≤25 and >25 kg/m^2^), or smoking status (ever smokers or never smokers). We also performed interaction tests between smoking and BMI with WC using a multiplicative model.

### Sensitivity analyses

In sensitivity analyses, we excluded the first 2 years of follow-up in order to decrease the potential bias of reverse causation. We also analyzed cohort participants whose age at recruitment was equal or younger than 60 years separately (data not shown). The overall results based on the aforementioned approaches were similar to the main analyses and did not change the overall interpretation of the results.

The proportional hazards assumption was tested on the basis of Schoenfeld residuals. Except for sex and center, which were included in the stratified analysis, all of the variables fitted the proportionality assumption. Two-sided tests with a significance level (*α*) of 0.05 were chosen. All analyses were performed using SAS 9.3 for Windows (SAS Institute Inc., Cary, NC, USA).

## Results

### Basic characteristics

During an average of 13.9 years of follow-up, 131 incident SICs were identified. Among them, the SIC cases were comprised of 41 adenocarcinomas, 44 carcinoids, and 46 other histological types (15 sarcomas, 10 lymphomas, and 21 unknown histology). Of the 131 SIC cases, 59 (45 %) were men and 72 (55 %) were women (Table 1). The average age at study entry was 56.6 years for cases and 51.8 years for non-cases. The distribution of education, smoking status, alcohol drinking, physical activity, family history of colorectal cancer, and comorbidity are given in Table 1. Briefly, cases tended to be less educated (41 % in the lowest education category compared to 35 % of non-cases), less physically active (28 % of cases versus 23 % of non-cases were inactive), and had marginally higher proportions of self-reported gastrointestinal comorbidities (i.e., gallstones, ulcer, and diabetes), whereas alcohol consumption in cases and non-cases was similar (average 7.2, 6.8 g/day, respectively).

**Table 1:** Characteristics of small intestinal cancer cases and cohort members in EPIC

Variables	Cases (*n* = 131)	Non-cases (*n* = 338,635)
Sex, *n* (%)		
Men	59 (45.0)	118,797 (35.1)
Women	72 (55.0)	219,838 (64.9)
Age at recruitment, years (mean, SD)	56.6 (8.5)	51.8 (10.1)
Age groups, years (*n*, %)		
<50	28 (21.4)	130,485 (38.5)
50–59	58 (44.3)	134,441 (39.7)
≥60	45 (34.4)	73,709 (21.8)
Education (*n*, %)		
None/primary school	54 (41.2)	119,433 (35.3)
Technical/professional school	29 (22.1)	84,158 (24.9)
Secondary school	20 (15.3)	52,907 (15.6)
Longer education (including university)	28 (21.4)	82,137 (24.3)
Smoking status (*n*, %)		
Never smoker	52 (39.7)	162,101 (47.9)
Former smoker	41 (31.3)	94,619 (27.9)
Current smoker	38 (29.0)	81,915 (24.2)
Alcohol drinking, g/day (median, P25–P75)	7.2 (1.1, 21.6)	6.8 (1.2,17.8)
Physical activity (*n*, %)		
Inactive	37 (28.2)	77,645 (22.9)
Moderately inactive	41 (31.3)	114,332 (33.8)
Moderately active	24 (18.3)	78,378 (23.2)
Active	29 (22.1)	68,280 (20.2)
Comorbidity (*n*, %)		
Diabetes	5 (3.8)	10,117 (3.0)
Gallstones	8 (6.1)	19,655 (5.8)
Cardiovascular diseases^a^	17 (13.0)	59,248 (17.5)
Allergic diseases^b^	12 (9.2)	34,763 (10.3)
Ulcer diseases	6 (4.6)	17,070 (5.0)

*SD* standard deviation; *P25* 25th percentile, *P75* 75th percentile

^a^Cardiovascular diseases: angina, heart diseases, stroke, and hypertension

^b^Allergic diseases: asthma, eczema, and other allergic diseases

### Height, weight, and BMI

SIC cases tended to be heavier (74.9 ± 15.3 kg) than non-cases (71.6 ± 13.8 kg) and to have a higher mean BMI (26.1 kg/m^2^) than non-cases (25.9 kg/m^2^; Table 2). Height and weight were associated with a slightly increased risk of SIC (multivariable HR per cm = 1.04, 95 % CI 1.00, 1.07; multivariable HR per kg = 1.01, 95 % CI 1.00, 1.03, Table 3). Overall, there was no association between BMI and SIC in the multivariable model (HR per kg/m^2^ = 1.00, 95 % CI 0.94, 1.05; Table 3) nor in the multivariable model that also included WC (HR per kg/m^2^ = 0.92, 95 % CI 0.84, 1.02). Similar results were observed for adenocarcinoma and carcinoids of the small intestine (Table 3). The association of height, weight, and BMI with SIC did not differ by subgroups of sex, BMI, or smoking status (Table 4).

**Table 2:** Anthropometric measures among small intestinal cancer cases (*n* = 131) and non-cases (*n* = 338,635)

	Cases		Non-cases	
	Mean (SD)	Median (IQR)	Mean (SD)	Median (IQR)
Height (cm)	168.9 (9.8)	168.7 (14.6)	166.1 (9.3)	165.4 (12.9)
Weight (cm)	74.9 (15.3)	73.5 (19.9)	71.6 (13.8)	70.0 (18.7)
Body mass index (BMI, kg/m^2^)	26.1 (4.3)	26.0 (5.5)	25.9 (4.4)	25.4 (5.5)
Hip circumference (cm)	102.3 (8.9)	101.0 (11.0)	101.0 (8.6)	100.0 (10.5)
Waist circumference (cm)	88.4 (14.1)	89.0 (21.1)	85.5 (13.1)	85.0 (19.5)
Waist-to-hip ratio	0.9 (0.1)	0.9 (0.2)	0.8 (0.1)	0.8 (0.1)
Waist-to-height ratio	0.5 (0.1)	0.5 (0.1)	0.5 (0.1)	0.5 (0.1)

*SD* standard deviation, *IQR* interquartile range

**Table 3:** HRs and 95 % CIs for small intestinal cancer risk in relation to anthropometric characteristics

	Total (*n* = 131)		Adenocarcinoma (*n* = 41)	Carcinoids (*n* = 44)
	Crude model	Multivariable model^a^	Multivariable model^a^	Multivariable model^a^
	HR (95 % CI)	HR (95 % CI)	HR (95 % CI)	HR (95 % CI)
Height (cm)	1.03 (1.00, 1.06)	1.04 (1.00, 1.07)	1.03 (0.96, 1.10)	1.08 (1.02, 1.15)
Weight (kg)	1.01 (1.00, 1.03)	1.01 (1.00, 1.03)	1.01 (0.98, 1.05)	1.03 (1.01, 1.06)
Body mass index (BMI, kg/m^2^)				
Not adjusted for WC	1.02 (0.97, 1.06)	1.00 (0.94, 1.05)	1.02 (0.92, 1.13)	1.05 (0.97, 1.15)
Adjusted for WC	0.94 (0.86, 1.01)	0.92 (0.84, 1.02)	0.84 (0.69, 1.01)	1.02 (0.87, 1.20)
Waist circumference (WC, per 5-cm increase)				
Not adjusted for BMI	1.08 (1.00, 1.17)	1.04 (0.94, 1.16)	1.17 (0.98, 1.40)	1.12 (0.94, 1.34)
Adjusted for BMI	1.20 (1.04, 1.39)	1.18 (0.98, 1.42)	1.56 (1.11, 2.17)	1.08 (0.78, 1.50)
Hip circumference (per 5-cm increase)				
Not adjusted for BMI	1.01 (0.88, 1.15)	1.09 (0.93, 1.28)	1.13 (0.89, 1.44)	1.19 (0.96, 1.48)
Adjusted for BMI	1.05 (0.86, 1.28)	1.10 (0.88, 1.37)	1.24 (0.87, 1.77)	1.23 (0.89, 1.69)
Adjusted for BMI + WC	0.99 (0.83, 1.19)	0.96 (0.77, 1.20)	0.99 (0.66, 1.50)	1.19 (0.82, 1.73)
Waist-to-hip ratio (percentage increase)				
Not adjusted for BMI	1.02 (1.00, 1.05)	1.02 (0.99, 1.05)	1.05 (0.99, 1.12)	1.01 (0.95, 1.07)
Adjusted for BMI	1.02 (0.99, 1.05)	1.02 (0.99, 1.06)	1.05 (0.99, 1.11)	1.00 (0.93, 1.07)
Waist-to-height ratio (percentage increase)				
Not adjusted for BMI	1.02 (0.99, 1.04)	1.00 (0.97, 1.04)	1.05 (0.99, 1.11)	1.01 (0.95, 1.08)
Adjusted for BMI	1.02 (0.98, 1.07)	1.02 (0.97, 1.07)	1.10 (1.00, 1.21)	0.98 (0.88, 1.08)

*HR* hazard ratio, *95* *% CI* 95 % confidence interval, *WC* waist circumference, *BMI* body mass index

^a^Adjusted for age, education, smoking status, alcohol drinking, physical activity, and/or anthropometrics when appropriate, stratified by sex and country

**Table 4:** HRs and 95 % CIs for small intestinal cancer risk in relation to anthropometric characteristics by sex, BMI, or smoking status

Variables	Sex		BMI		Smoking	
	Male (59 cases)	Female (72 cases)	≤25 (54 cases)	>25 (77 cases)	Ever smokers (79 cases)	Never smokers (52 cases)
	HR (95 % CI)^a^	HR (95 % CI)^a^	HR (95 % CI)^a^	HR (95 % CI)^a^	HR (95 % CI)^a^	HR (95 % CI)^a^
Height (cm)	1.05 (1.00, 1.10)	1.03 (0.98, 1.08)	1.01 (0.97, 1.05)	1.03 (1.00, 1.07)	1.02 (0.98, 1.05)	1.04 (0.99, 1.08)
Weight (kg)	1.02 (0.99, 1.05)	1.00 (0.98, 1.03)	0.99 (0.95, 1.03)	1.02 (1.00, 1.04)	1.00 (0.98, 1.02)	1.02 (1.00, 1.05)
Body mass index (BMI, kg/m^2^)						
Not adjusted for WC	1.02 (0.93, 1.13)	0.99 (0.92, 1.06)	0.87 (0.73, 1.03)	1.01 (0.93, 1.10)	0.97 (0.90, 1.05)	1.03 (0.96, 1.11)
Adjusted for WC	0.89 (0.74, 1.06)	0.97 (0.85, 1.09)	0.85 (0.68, 1.06)	0.94 (0.84, 1.05)	0.89 (0.78, 1.01)	1.00 (0.89, 1.14)
Waist circumference (WC, per 5-cm increase)						
Not adjusted for BMI	1.12 (0.95, 1.32)	1.00 (0.87, 1.14)	0.93 (0.75, 1.15)	1.12 (0.98, 1.28)	1.02 (0.90, 1.15)	1.08 (0.93, 1.24)
Adjusted for BMI	1.35 (0.98, 1.86)	1.06 (0.83, 1.36)	1.04 (0.81, 1.34)	1.21 (1.00, 1.46)	1.19 (0.97, 1.46)	1.07 (0.84, 1.36)
Hip circumference (per 5-cm increase)						
Not adjusted for BMI	1.11 (0.87, 1.41)	0.97 (0.82, 1.15)	0.82 (0.60, 1.13)	1.04 (0.87, 1.25)	0.93 (0.77, 1.13)	1.10 (0.91, 1.32)
Adjusted for BMI	1.22 (0.88, 1.70)	0.96 (0.74, 1.24)	0.85 (0.59, 1.22)	1.10 (0.88, 1.38)	1.01 (0.77, 1.32)	1.08 (0.81, 1.44)
Adjusted for BMI + WC	0.97 (0.64, 1.45)	0.93 (0.71, 1.24)	0.85 (0.58, 1.24)	1.02 (0.81, 1.28)	0.92 (0.69, 1.23)	1.07 (0.79, 1.44)
Waist-to-hip ratio (WHR, percentage increase)						
Not adjusted for BMI	1.04 (0.98, 1.09)	1.01 (0.97, 1.05)	1.00 (0.96, 1.04)	1.03 (0.99, 1.06)	1.01 (0.98, 1.05)	1.01 (0.97, 1.05)
Adjusted for BMI	1.05 (0.99, 1.11)	1.01 (0.97, 1.06)	1.01 (0.96, 1.05)	1.03 (0.99, 1.06)	1.03 (0.99, 1.07)	1.00 (0.96, 1.05)
Waist-to-height ratio (WHtR, percentage increase)						
Not adjusted for BMI	1.02 (0.96, 1.08)	0.99 (0.95, 1.04)	0.95 (0.87, 1.04)	1.02 (0.97, 1.07)	1.00 (0.96, 1.05)	1.01 (0.96, 1.06)
Adjusted for BMI	1.06 (0.97, 1.16)	0.99 (0.92, 1.07)	0.96 (0.87, 1.06)	1.05 (0.99, 1.12)	1.05 (0.98, 1.13)	0.98 (0.91, 1.07)

*HR* hazard ratio, *95* *% CI* 95 % confidence interval, *BMI* body mass index

^a^Adjusted for age, education, smoking status, alcohol drinking, physical activity, and/or anthropometrics when appropriate, stratified by sex and country

### Waist circumference (WC) and hip circumference (HC)

WC was marginally higher among cases (88.4 cm) compared with non-cases (85.5 cm; Table 2). In models adjusted for BMI, WC was positively associated with SIC in the crude model (HR per 5 cm = 1.20, 95 % CI 1.04, 1.39) but this association attenuated in the multivariable model (HR per 5 cm = 1.18, 95 % CI 0.98, 1.42; Table 3). By histological subtype, WC was statistically significantly associated with adenocarcinoma (multivariable HR adjusted for BMI = 1.56, 95 % CI 1.11, 2.17), but not with carcinoid tumors (HR 1.08, 95 % CI 0.78, 1.50; Table 3). In stratified analyses, the association between WC and SIC did not differ by sex or smoking status, but it was stronger for those with a BMI > 25 kg/m^2^ (Table 4).

We did not observe any statistically significant associations between HC and SIC overall or by histological subtypes, sex, or smoking status (Tables 3, 4).

### Waist-to-hip ratio (WHR) and waist-to-height ratio (WHtR)

A marginally positive association was observed for WHR and SIC (crude HR 1.02, 95 % CI 1.00, 1.05; multivariable HR 1.02, 95 % CI 0.99, 1.05); additional adjustment for BMI did not change the results (Table 3). The results for WHtR were similar to WHR and revealed a positive association with SIC (Table 3) that was more evident for adenocarcinomas of the small intestine (multivariable HR 1.10, 95 % CI 1.00, 1.21; Table 3). No further significant results were found in the stratified analyses by sex, BMI groups, and smoking.

## Discussion

The current study suggests that abdominal obesity rather than overall obesity might be associated with an increased risk of adenocarcinoma of the small intestine; however, these associations are based on a small number of cases.

The strengths of the current study include the large population-based cohort design with a long follow-up period of around 14 years, where exposure data were collected at baseline prior to cancer detection. Baseline data on relevant confounders such as physical activity, smoking, alcohol drinking, education, and diet were also available. In addition, incident cancers and deaths were retrieved through linkage to health registries or medical records in the different EPIC centers. There are, however, also weaknesses of the current study. Specifically, the small number of cases in our study limited the analyses and interpretability of findings, particularly within histological subgroups, as well as the statistical power to detect associations. The small sample size also limited our ability to investigate a full range of potential confounders; for example, we were unable to address diabetes as a potential confounder because only five cases reported having diabetes and we did not have information on fasting glucose levels on the cohort. Given these weaknesses, our results should be interpreted with caution and further studies with a larger number of cases are warranted. However, this currently remains one of very few cohort studies that have investigated risk factors for SIC.

Two previous register-based studies demonstrated a positive association between obesity and SIC mainly in men. The Swedish register-based study showed that the relative risk of SIC among obese men was 4.0 (95 % CI 2.2–9.3), whereas the relative risk of SIC in women was 1.9 (95 % CI 0.8–3.7) [24]; however, only 17 SIC cases were included in this study. In the US veterans study, obesity was associated with an increased risk of SIC in white men but not in black men [25]. The definition of obesity in these two studies was based on clinical diagnosis (BMI ≥ 40 kg/m^2^), which might underestimate the real association between generally defined obesity (BMI ≥ 30 kg/m^2^) and SIC. Similar results with high BMI in men were also indicated in the Norwegian health survey study [27]. However, information such as more detailed anthropometric measurements, physical activity, smoking, and alcohol drinking was lacking in all of these studies. In a pooled cohort study among Asian populations, no significant association was found between SIC and BMI, although there was a suggestive positive association among men [28]. In the NIH-AARP Diet and Health study, which included more than half a million participants, high BMI was associated with an increased risk of malignant carcinoid tumors but not adenocarcinomas of the small intestine [21]. However, in one small case–control study from Italy, individuals with a BMI less than 20 kg/m^2^, compared to those with a BMI greater than 20 kg/m^2^, had an increased risk of SIC (odds ratio 4.58, 95 % CI 1.48–14.16) [20]; none of these previous studies examined the association between abdominal obesity and SIC.

Abdominal obesity has been positively associated with other gastrointestinal cancers, including colorectal cancer [37, 38] and esophageal adenocarcinoma [39, 40], but no epidemiologic studies have reported the association between abdominal obesity, assessed by WC, WHR, HC, or WHtR, and risk of SIC. Although we have a relatively small number of SIC cases in the current study, our results indicate potentially differential risk estimates for general obesity (as measured by BMI) and abdominal obesity. BMI may not be a perfect measure for adiposity because it does not differentiate body fat from muscle mass [36]. Therefore, we analyzed the association of other anthropometric indices with risk of SIC considering several models to assess different biological interpretations of overall obesity, lean body mass, and abdominal obesity. In the model including residuals of WC and BMI [36], WC residuals reflect abdominal adiposity, while BMI represents overall adiposity. In the model with WC not adjusted for BMI, WC represents abdominal obesity that may be confounded by BMI. Results from both of the models indicated abdominal obesity rather than overall obesity was positively associated with SIC. In contrast, in a model with WC (not WC residuals) and BMI, WC would still reflect abdominal adiposity, but BMI would probably be more a measure of lean body mass since body fatness is to a large extent accounted for by WC, especially in older adults [36]; however, our study did not detect statistical heterogeneity between models with adjustment for residuals or not, perhaps due to a limited number of cases.

The role of obesity in SIC could be complex, and in our study abdominal obesity seems to play a more important role compared to overall obesity, specifically for adenocarcinoma of the small intestine. Gastrointestinal adenocarcinomas have been associated with abdominal obesity in accumulating studies, while the etiology of gastrointestinal carcinoids might be different. Several possible biological mechanisms may explain the association between abdominal obesity and adenocarcinoma of the small intestine. First, individuals with abdominal obesity are generally viscerally obese, which may reduce the movement of the small intestine; the physically active motility of the small intestine has been regarded as one of the reasons for the rarity of SIC [5]. Second, intra-abdominal obesity promotes insulin resistance, a state of reduced responsiveness of tissues to the physiologic actions of insulin [41]. Obese individuals, especially those with abdominal obesity, often have increased levels of insulin and insulin-like growth factor-1 (IGF-1), which may promote the development of SIC, as has been hypothesized for other gastrointestinal tumors [42]. Third, some studies have reported higher leptin levels among lean individuals with abdominal obesity compared with those with overall obesity [43–45]. Leptin is suggested as a risk factor for colorectal malignancies [46, 47]. Leptin is derived from adipocytes and appears to play an important role in the regulation of ghrelin, a peptide derived from the stomach and small intestine that stimulates appetite and weight gain. Moreover, leptin seems to play diverse roles in the gastrointestinal tract including modulation of motility, absorption, and inflammation [43]. Other factors prominent potential mechanisms linking abdominal obesity to SIC include high levels of estrogen produced from fat tissue and chronic inflammation, as well as lower levels of adiponectin [48].

In summary, abdominal obesity was positively associated with adenocarcinoma of the small intestine but not with malignant carcinoid tumors. Although suggestive, these findings should be interpreted with caution due to the small number of cases by histological subtype. Further investigation using pooled data from multiple cohort studies to generate a larger sample of SIC cases is warranted.
